# Setup, Test and Validation of a UHF RFID System for Monitoring Feeding Behaviour of Dairy Cows

**DOI:** 10.3390/s20247035

**Published:** 2020-12-09

**Authors:** Felix Adrion, Markus Keller, Giulia Bianca Bozzolini, Christina Umstatter

**Affiliations:** Agroscope, Research Division Competitiveness and System Evaluation, Research Group Automation and Labour Organisation, Tänikon 1, CH-8356 Ettenhausen, Switzerland; markus.keller@agroscope.admin.ch (M.K.); giuliabianca.bozzolini@agroscope.admin.ch (G.B.B.); christina.umstaetter@agroscope.admin.ch (C.U.)

**Keywords:** eating time, bout criterion, transponder, antenna

## Abstract

Feeding behaviour can be used as an important indicator to support animal management. However, using feeding behaviour as a tool for dairy cow management an automatic sensor system is needed. Hence, the objective of this study was to setup, test and validate a ultra-high frequency (UHF) radio-frequency identification (RFID) system for measuring time dairy cows spent at the feed fence using two types of passive UHF ear tags. In a first experiment, the reading area of the system was evaluated in two antenna positions. Subsequently, the UHF RFID system was validated with video observations and compared to the measurements of chewing time of a noseband pressure sensor and of the time spent at the feed fence registered by a sensor system with real-time localisation. Differences in the reading area were detected between the two antenna positions and types of ear tag. The antenna position leading to less false positive registrations was chosen for the experiment with cows. The validation with video data showed a high average sensitivity (93.7 ± 5.6%, mean ± standard deviation), specificity (97.8 ± 1.1%), precision (93.8 ± 2.3%) and accuracy (96.9 ± 0.9%) of the UHF RFID system for measuring the time spent at the feed fence. The comparison with the noseband pressure sensor and the real-time localisation resulted in high correlations with a correlation coefficient of *r* = 0.95 and *r* = 0.93, respectively. However, substantial absolute differences between the three systems pointed out differences between direct and indirect measures of feeding behaviour in general and between the different sensors in particular. Thus, detailed considerations are necessary before interpreting automatically measured feeding data generally.

## 1. Introduction

Feeding behaviour is among the most important behaviours of an animal and can be used as an indicator to support livestock management. Changes in feeding behaviour are not only caused by changes in feed or management, but especially by diseases or welfare impairments [1,2]. To detect changes in behaviour reliably, automated sensor systems are needed, that are able to record feeding behaviour. The most precise way to measure not only feeding time, duration and daily pattern but also feed intake, is the use of feed bunk weighing systems with electronic identification of single animals [3,4]. These systems are widely used in research, but are too expensive for use on commercial farms. Another way to record feeding and also rumination behaviour are sensor systems that either detect the jaw movements of the cow directly [5] or indirectly by measuring the movement of the head or neck [6] or ear [7]. However, these systems demand a power supply on the sensor. A possible solution for developing a feeding behaviour monitoring system could be radio-frequency identification (RFID) systems using passive transponders without internal power supply. Different RFID frequencies such as low-frequency (LF), high-frequency (HF) and ultra-high frequency (UHF) have been applied for this task [8,9]. Recently, researchers have applied especially UHF RFID technology for measuring different behaviours of pigs [10], poultry [11] and cattle [12]. Like LF RFID technology, which is commonly used for mandatory animal identification and also management applications, UHF RFID is based on passive transponders, which do not require a battery [13]. The advantages of UHF RFID in comparison to other RFID frequencies are a flexible and high reading range up to 12 m and the ability to detect over 100 animals per second. However, water and body tissue can have a strong influence on the reading range and directional pattern of UHF transponder antennas and also the reader antenna is strongly influenced especially by metal [14,15,16]. Thus, UHF RFID systems need to be adapted and tested thoroughly within new application environments. A detailed overview about properties of different RFID frequencies, validation procedures for RFID systems and the possibilities to use the data from these systems has been given by Brown-Brandl et al. [13].

It has to be emphasised that with all RFID systems only the presence of a tag (i.e., the animal) in the reading area of the antenna can be detected. The connection of the mere presence of the animal to its behaviour has to be made as close as possible by setting up the reading area in a way which makes it highly likely that the animal is performing the behaviour of interest at this place. For some behaviours like feeding this seems to be easier than for rather unspecific behaviours like grooming [10,12]. In some cases it might be possible to combine the RFID detections with other sensor signals, e.g., a water flowmeter at a drinker [17,18]. At the current stage, RFID systems for monitoring of group-housed animals in a commercial setting are mostly in the testing and evaluation phase. For some applications proper setups have been found, but especially with cattle, the potential of this technology still has to be scientifically evaluated.

The overall aim of a first experiment was to test and optimise the system by finding a setup that allowed for reliable identification of UHF ear tags in front of the feed fence with as few as possible detections behind the fence. The objectives of this experiment were (1) to setup a UHF RFID system for measuring time dairy cows spent at the feed fence using passive UHF ear tags and (2) to characterise the size and shape of the reading area of the system testing two different antenna positions and two types of UHF ear tags. In a further experiment, the objectives were (3) to validate the UHF RFID measurements of cows’ visits at the feed fence with video observations and (4) to compare the measurements to eating time measurements of a noseband pressure sensor and to time–space data of a sensor system with real-time indoor localisation.

## 2. Materials and Methods

To test and optimise the setup and functioning of the UHF RFID system, a first experiment was conducted. A part of Experiment 1 has already been published as a conference contribution [19]. After analysis of the data a decision for an antenna position was made, the system was completed and a second experiment including dairy cows was performed. Thus, all sections of the paper are split into Experiment 1, including the setup of the UHF RFID system and the measurements of the reading area (Objectives 1 and 2) and Experiment 2, including the validation and comparison of the UHF RFID system with two other sensor systems (Objectives 3 and 4).

All measurements were carried out in an experimental dairy barn for comparative emission measurements at BBZ Arenenberg in Tänikon (Switzerland) described by Schrade et al. [20]. The barn is divided into two compartments for 20 cows each. Both experiments of this study were conducted in the northern compartment of the barn. The second experiment included 10 dairy cows. All animal experimental procedures were approved by authorities of Canton Thurgau, Switzerland (authorisation no. TG 02/17) in accordance with Swiss animal protection legislation.

### 2.1. Experiment 1: Setup and Test of the UHF RFID System

#### 2.1.1. UHF RFID Equipment

At the feed fence (Comfort Flexi, Krieger AG, Lenggenwil SG, Switzerland) of the barn compartment a free-form UHF cable antenna (Locfield^®^ Antenna, Cavea Identification GmbH, Olching, Germany) was mounted. This type of antenna was chosen because of the possibility of covering a relatively long part of the feed fence with only one antenna. The antenna had an active length of 6 m and a passive length of 1 m including the damping unit made of ferrites. The main component of the antenna was a coaxial cable with 5 mm diameter and an attenuation of approx. 0.29 dB/m. It was connected to a UHF reader with a coaxial cable of 6 m length damping 0.29 dB/m (‘H-155 PE’, Belden Wire & Cable B.V., Venlo, The Netherlands). In total, this resulted in a loss of approx. 3.8 dB from the reader to the tip of the antenna, where the antenna field originated.

The reader used was an Impinj Speedway Revolution R420 model for the European Telecommunications Standards Institute (ETSI) frequency range (Impinj Inc., Seattle, WA, USA). The output power of the reader was set to 31.5 dBm during the tests, resulting in an effective output power of approx. 27.7 dBm at the tip of the cable antenna. The receiver sensitivity was set to the maximum value of 70 dBm and the reader could flexibly switch between Channels 4, 7, 10 and 13 (865.7, 866.3, 866.9 and 867.5 MHz). Additionally, the reader operated in “dense reader mode (*M* = 8)” to enhance the robustness against other activity within the UHF RFID frequency range. Data acquisition was performed with the Software Multireader (Version 6.6.13, Impinj Inc., Seattle, WA, USA).

**Figure 1:**
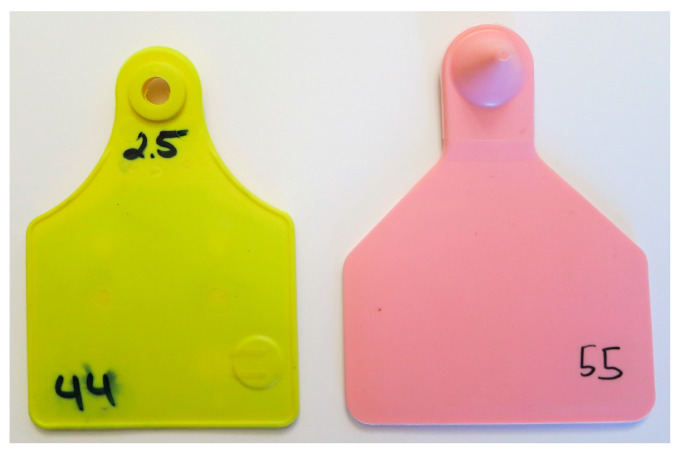
(**left**): ultra-high frequency (UHF) ear tag Type A, (**right**): UHF ear tag Type B.

The tests were conducted with two different types of UHF transponder ear tags, which both were functional models and were not commercially available. Type A contained a transponder with a planar inverted F-shaped (PIF) antenna approx. 50 × 50 mm^2^ in size (deister electronic GmbH, Barsinghausen, Germany). It was combined with an Impinj Monza^®^ 4D chip (Impinj Inc., Seattle, WA, USA) and integrated into a Primaflex^®^ cattle ear tag (Caisley International GmbH, Bocholt, Germany) by injection molding (Figure 1). Type B was a functional model provided by Scot EID Livestock Traceability Research (Huntly, United Kingdom). It contained an Alien Garment Tag (GT) (ALN-9728, Alien Technology LLC, San José, CA, USA) with a dipole antenna and an Alien Higgs^®^ 4 chip. This tag was 50 × 30 mm^2^ in size. It was encapsulated in an air-filled pocket moulded onto a regular two-piece ear tag for cattle.

#### 2.1.2. Installation of the UHF RFID Antenna

Since the method of mounting and the proximity to surrounding materials strongly influence the antenna field and the reading performance of UHF RFID antennas, the experimental setup is subsequently described in detail.

**Figure 2:**
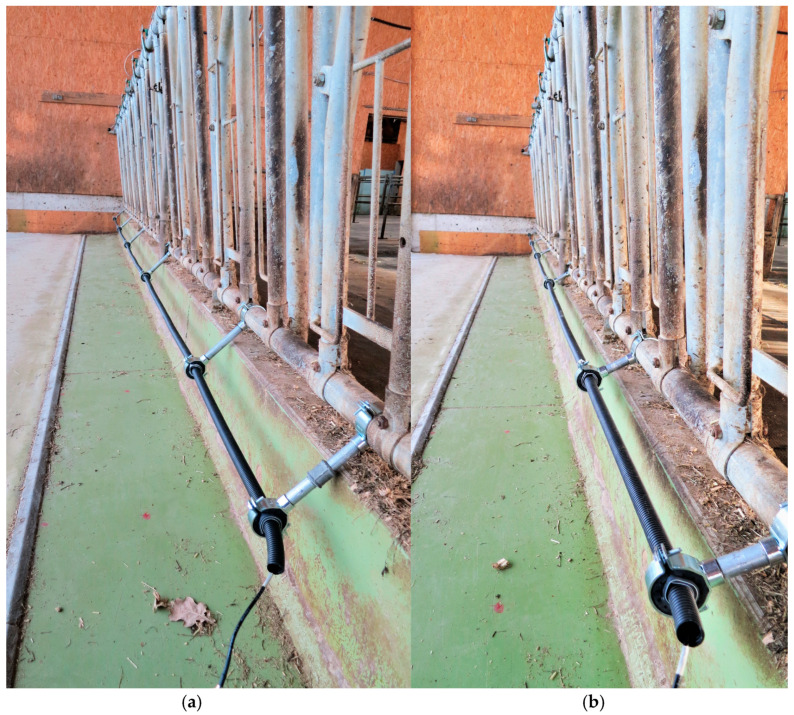
Setup of the antenna at the feed fence. (**a**) Antenna Position 1; (**b**) Antenna Position 2.

Two different mounting positions of the antenna were tested (Figure 2). Since pre-tests showed that the antenna field was weakened severely in proximity to reinforced concrete or metal, the antenna was mounted with a distance to the feed fence and the wall below the fence. For this purpose, six paired pipe clamps (clamp diameter 2 inch at the fence, 1 inch at the antenna) were mounted to the feed fence and connected with a metal pipe (diameter 0.5 inch). The first pair of clamps was mounted at a distance of 26 cm to the left side wall of the barn compartment. The other clamps followed along the feed fence at distances of 179, 157, 161, 150 and 68 cm to fit in between the feeding places. The 1” clamps were filled with a rubber foam to hold a flexible polyvinyl chloride (PVC) pipe (PLICA UV-FLEX M25, Plica AG, Frauenfeld, Switzerland). The antenna was inserted into the flexible PVC pipe, so that it was mechanically protected and could not touch the metal clamps.

The reinforced concrete wall below the feed fence was 14 cm wide. The top of the wall was 33 cm above the feeding alley and 48 cm above the walking alley behind the feed fence. Two antenna positions were tested. In Position 1, the antenna was mounted in a horizontal distance of 18 cm to the centre of the wall and 24 cm above the concrete floor of the feeding alley. In Position 2, the horizontal distance to the centre of the wall was reduced to 14 cm and the height above the floor was increased to 32 cm. This antenna position was chosen as an alternative to Position 1, because it would interfere much less with the movement of the cows’ head during feeding. The length of the metal pipe connecting the two parts of each pair of pipe clamps was 16 cm and 8 cm for Antenna Positions 1 and 2, respectively (see Figure 2).

The tip of the cable antenna was placed at 63 cm distance from the left side wall of the barn compartment. In this way, the antenna started slightly before Feeding Place 1 covering 8 feeding places in total. Its active length ended 30 cm behind Feeding Place 8. The middle of the first feeding place of the fence was at a distance of 83 cm from the left side wall. The further places followed in an interval of approx. 78 cm (±1 cm). The headlocks of the feeding places were set to the “closed” position for this experiment to achieve similar conditions along the antenna.

#### 2.1.3. Measurements of the Reading Area

**Figure 3:**
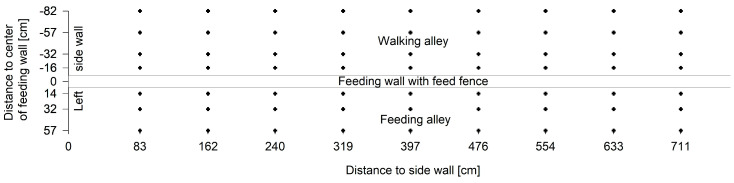
Coordinate grid for reading range measurements in front and behind the feed fence. Measurements were conducted at heights of 30, 55, 80 and 105 cm above the floor. Each x-coordinate represents the centre of a feeding place along the feed fence.

A coordinate grid was established to measure the reading range of the antenna in front of and behind the feed fence (Figure 3). In the middle of each of the first 9 feeding places measurement points were marked close to the feeding wall on both sides (+14 cm (front) and −16 cm (back) from the centre of the wall) and in greater distances (+32, +57, −32, −57 and −82 cm). The coordinates in front of the feed fence represented the middle and end of the floor coating, where the feed for the cows is normally provided. At the backside of the feed fence, the measurement points at −82 cm were added to detect unwanted readings in this area. Pre-tests showed that beyond this point no ear tags could be detected in both antenna positions. The measurements according to the coordinates were repeated in four different heights above the floor on both sides of the feed fence (30, 55, 80 and 105 cm). The bottom of the tags was positioned in these heights for the measurements.

**Figure 4:**
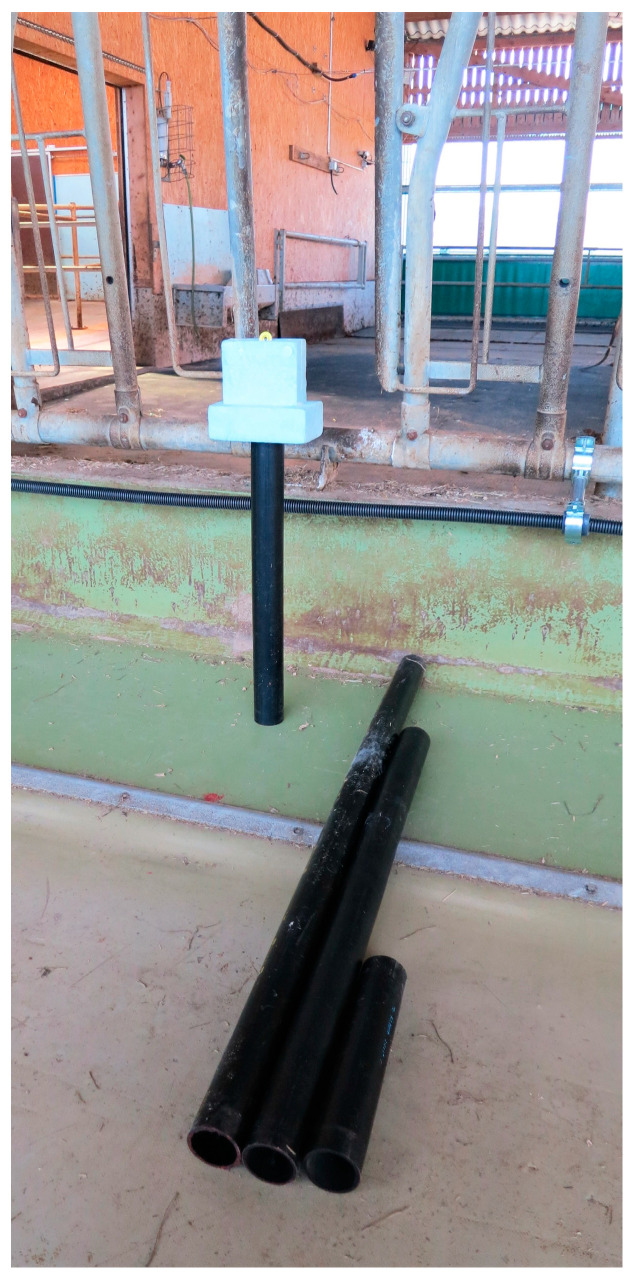
Polystyrene holder with ear tag and polyvinyl chloride (PVC) pipes of different lengths to maintain the targeted height during the measurements.

Therefore, an ear tag holder was constructed out of polystyrene foam to ensure that all measurements were performed identically, at the correct height, and with as little influence of the measuring person on the tags as possible (Figure 4). Polystyrene foam has very little influence on the electromagnetic fields of the reader and tag (relative permittivity ε_r_ = 1.03) and is used as a standard mounting material in high-frequency tests. The tags were inserted in a vertical position into a notch in the material. A hole was drilled into the bottom of the holder to be able to put it on top of PVC pipes. Four PVC pipes of different lengths with a diameter of 5 cm held the tags in the targeted heights above the floor. The distance between the top of the pipes and the bottom of the tag in the holder was 4 cm ensuring little influence of the PVC on the tags. For the measurements, the holder with the ear tag was positioned at a coordinate and then turned around 360° horizontally. If at least one successful reading of the tag took place during this turn, the tag was labelled as “detected” at this coordinate.

Three samples of each of the two types of UHF ear tags were randomly chosen for the experiment. Out of these samples, three pairs with one tag of each type were combined randomly and the measurement order within these pairs was also randomly chosen. The pairs were measured one after each other. First, all measurements with Antenna Position 1 were conducted. All measurements of each UHF ear tag were performed at once before measuring the next tag. The order of the measurements was the following: starting at the closest coordinate to the feeding wall at the front of the feed fence (+14 cm) at the first feeding place, first, the distance to the feeding wall was increased by proceeding to the next coordinates (+32 cm, +57 cm). This was done for each of the 9 feeding places one after each other. After finishing all measurements at one height, the measurements at the next height level were conducted identically. After measuring at the coordinates at the front of the feed fence the same procedure was conducted at the coordinates at the back of the feed fence. The same procedure was followed with Antenna Position 2 with the same, but newly randomised ear tags. It has to be noted that at a height of 30 cm no measurements could be made directly in front of the feed fence (horizontal position +14 cm), because the antenna was blocking this position in both treatments.

#### 2.1.4. Visualisation of the Reading Area

For the data analysis of this experiment, the number and percentage of successful detections for each coordinate was calculated per type of tag and antenna position for each height separately and over all heights. At maximum, 12 detections were achievable during the measurements of three tags in four heights (only three heights at the coordinate +14 cm), resulting in a detection rate of 100%. With these data, contour plots were created using Sigmaplot 13 (Systat Software Inc., San José, CA, USA) for graphical analysis of the data. Furthermore, the number of coordinates with at least one successful detection over all heights in front of and behind the feed fence was calculated for each antenna position and type of ear tag.

### 2.2. Experiment 2: Validation of the UHF RFID System and Comparison to Two other Sensor Systems

#### 2.2.1. Installation and Technical Setup of the Complete UHF RFID System

**Figure 5:**
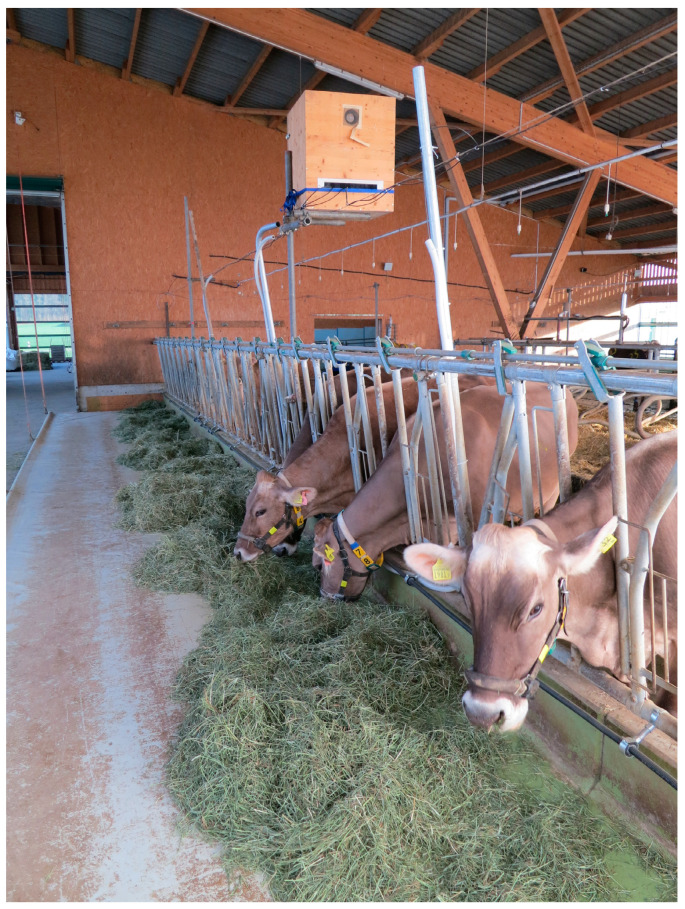
Installation of the three cable antennas along the feed fence.

After completing the first experiment, Antenna Position 2 was chosen for the setup of the system along all 20 feeding places, minimising the number false positive readings behind the feed fence (see discussion in Section 4.1). In total, three identical cable antennas of 6 m active length were mounted in a similar way as the first antenna was installed for the first experiment. The same PVC pipe and the same pipe clamps were used to mount the antennas. To join all coaxial cables at the reader, Antennas 1 and 2 were installed with the tip towards the left side of the feed fence, and Antenna 3 was installed with the tip towards the right side of the feed fence. The reader was mounted in a ventilated wooden box above the feed fence between Feeding Place 13 and 14. The first antenna was installed beginning at the first feeding place, 0.71 m distant to the left end of the feed fence. Antenna 2 started at 6.25 m distance from the left end and Antenna 3 went backwards from 15.55 m distance to the left end (0.6 m distance to the right end) to a distance of 9.90 m from the left end of the feed fence. The last 0.35 m of the active length of Antenna 3 then went upwards towards the reader. Pre-tests showed that this did not result in a larger reading field at the back of the feed fence. The length of the 3 antennas (18 m total length) exceeded the length of the feed fence (16.15 m). Hence, and to compensate for the weak reading field at the back part of the antennas, Antenna 1 and 2 were installed with an overlap of 0.46 m and Antenna 2 and 3 with an overlap of 2.35 m (Figure 5). The overlap of Antenna 2 and 3 was much larger, since these two antennas overlapped with their back parts.

Each antenna was mounted with five pairs of pipe clamps. The clamps for Antenna 1 were mounted at a distance of 0.36, 1.92, 3.48, 5.04 and 6.60 m. The clamps for Antenna 2 were at 5.91, 7.29, 8.16, 9.72 and 11.28 m. Antenna 3 was held by clamps at 10.51, 11.92, 13.10, 14.40 and 15.96 m distance, all measured from the left end. The pipe clamps were all installed at the same height and distance to the feeding wall, except for the rightmost clamp of Antenna 1 and the leftmost two clamps of Antenna 3. These were installed 1 cm lower at 0.31 m distance to the ground and 1 cm closer to the centre of the wall (13 cm). This was necessary to allow one antenna pass below the other at the overlap.

The RFID equipment, i.e., antennas, reader, software, coaxial cables and ear tags were the same as in the first experiment. The settings of the reader were also kept the same. The antennas were multiplexed automatically by the reader. The inventory settings of the reader were set to search mode “Single Target Inventory” and “Session 1”. With the settings chosen, approx. 1–2 readings per second were possible with ear tag Type A and 2–4 readings with ear tag Type B. The experiment was performed with 10 lactating Brown Swiss dairy cows between first and fourth lactation (mean of 2 lactations). Half of the group was tagged with ear tag Type A and the other half was tagged with ear tag Type B. All animals were tagged in their left ear. Specifications of the UHF RFID system not mentioned here can be found in Section 2.1.1.

#### 2.2.2. Systems for Validation and Comparison of the UHF RFID System

Experiment 2 was split into two parts, a validation part comparing the UHF RFID data with video observations and a second part consisting of a comparison between three sensor systems, the UHF RFID system, a sensor system with a noseband pressure sensor and a sensor system with real-time localisation. It should be noted that the data of the three sensor systems used in this study represent different aspects of feeding behaviour.

The video observations were performed with a Mobotix M 15 D-Sec. Camera (Mobotix AG, Langmeil, Deutschland), which was mounted above and in front of the left end of the feed fence. The camera looked down and along the feed fence in a wide angle, so that the whole feed fence was visible in the video image. The video data were recorded with MX-V4.4.2.73 and MxManagementCenter 2.1.11.0 software (Mobotix AG).

All cows were equipped with a RumiWatch halter (RumiWatchSystem, Firmware version 02.23, Itin + Hoch GmbH, Liestal, Switzerland) to measure the eating time. This system utilises a pressure tube in the noseband of the halter to measure the jaw movements when the animal is chewing and calculates the eating and rumination time separately from this data. Details about the function and accuracy of the system have been shown by Zehner et al. [5].

In addition, all cows were equipped with a sensor ear tag of the Smartbow^®^ system (Smartbow GmbH/Zoetis Services LLC, Weibern, Austria). This system measures the location of the ear tags in real-time by using the Time Difference of Arrival principle. For this, 7 receiver antennas were mounted on the walls of the northern barn compartment. The operating frequency of the system is 2.4 GHz. The minimum position error of the system was estimated at approximately 1.2 m by Wolfger et al. [21]. For the purpose of measuring the time spent at the feed fence, an area along the feed fence, extending from approx. 0.4 m behind to 1.6 m in front of the feed fence, was defined in the system software. In this way, the visiting time of the cows in this area could be extracted from the data.

#### 2.2.3. Validation Experiment with Dairy Cows

The experiment was performed over a period of 6 days in May 2019. Cows were on pasture during most days, usually between 07:00:00 and 16:30:00, depending on weather conditions. Thus, the data collection for validation and comparison of the system took place during different time periods on the test days. For the validation with video data, 09:54:38 h of data (12:00:00 to 21:54:38) on Day 1 were analysed. For the comparison of the UHF system with the RumiWatch system and the Smartbow^®^ system, 12 h on Day 1 (12:00:00 to 23:59:59), 16 h on Day 2 (08:00:00 to 23:59:59) and 5 h each on Days 3 to 6 (19:00:00 to 23:59:59) were analysed, resulting in a total of 48 h.

#### 2.2.4. Data Preparation and Analysis

Video observations were used as a control for validating the UHF system in this study. Manual labelling of the cows’ time spent at the feed fence was performed using the software MEZA 8.8.21 (Drigus Systeme GmbH, Dortmund, Germany). One person labelled all video data, ensuring the same labelling procedures were applied throughout. It was not recorded if the cows were actually feeding as the RFID system could only determine whether a cow’s UHF ear tag was within the reading area of the antenna, but not whether the cow was feeding or not. A cow was defined as “within the target area” when she put her head through the feed fence at any of the 20 feeding places (visiting event). In addition, it was determined, whether her head was up or down during the time she spent within the target area (sub-event). As soon as a cow pulled her head out of the feed fence, she was labelled as “out of the target area” (non-event). For each visiting event time stamps of the beginning and end of the event were recorded. Two datasets were created from the raw labelling data using a Visual Basic for Applications Macro in Excel 2016 (Microsoft Corporation, Redmond, WA, USA). In the first data set, all visiting events including both sub-events “head up” and “head down” were defined as visiting events. In the second data set, only the sub-events “head down” were defined as visiting events. This was done to examine, if the UHF RFID system was detecting the cows with both head positions or only with head down. The macro automatically joined and/or divided all adjacent events to new visiting events and non-events in both data sets.

The UHF RFID data were recorded as individual registrations of the ear tags containing the individual ID of the tag and the time stamp of the registration. The ear tags were mostly detected in irregular intervals, since their movement relative to the cow’s ear and within the reading field led to changing reading performance, which is typical for UHF RFID tags [14]. To fill in the resulting reading gaps, the raw registrations had to be aggregated to visiting events. For this, a bout criterion was applied as described in Brown-Brandl et al. [13]. A bout criterion is the maximum allowed interval between two readings of the same tag so that both readings are part of the same visiting event. Several different bout criteria of 60, 80, 100, 120, 150, 180 and 300 s were applied to the UHF data for validation with both video data sets. The bout criterion resulting in the maximum average accuracy and the minimum number of false positives and false negatives of the UHF data of all cows with both types of tags was chosen.

The accuracy was determined together with the sensitivity, specificity and precision (positive predictive value) according to common definitions for both types of tag together and separate (Equations (1)–(4)) [9,10]. The analyses were done on the level of seconds, again using a Visual Basic for Applications Macro in Excel 2016. For the calculation of the performance measures, the macro labelled all seconds of the validation time true positive, false positive, true negative or false negative according to the following definitions:

▪ Positives (P): s during which a cow was observed in the target area.
▪ Negatives (N): s during which a cow was not observed in the target area.
▪ True positives (TP): positives which were part of an RFID event of the same cow.
▪ False positives (FP): negatives which were part of an RFID event of the same cow.
▪ True negatives (TN): negatives which were not part of an RFID event of the same cow.
▪ False negatives (FN): positives which were not part of an RFID event of the same cow.

(1) $$Sensitivity\;\left[\%\right]=\frac{TP}{P}\times 100$$

(2) $$Specificity\;\left[\%\right]=\frac{TN}{N}\times 100$$

(3) $$Precision\;\left[\%\right]=\frac{TP}{\left(TP+FP\right)}\times 100$$

(4) $$Accuracy\;\left[\%\right]=\frac{\left(TP+TN\right)}{\left(P+N\right)}\times 100$$

For the comparison of the UHF RFID system with the RumiWatch noseband sensor (eating time data) and the Smartbow^®^ system (location data), hourly data of all three methods were used. Since both types of tags achieved similar results in the validation, the measurements of both were combined in this analysis. The durations of the UHF RFID visits were summed up for each cow in each of the 48 h analysed. The RumiWatch raw data was converted with the RumiWatch converter Version 0.7.4.13. The system calculates the eating time from the measured jaw movements and distinguishes between feeding with head up and head down. Since there is no option to distinguish between head up and down with the UHF RFID system, the two categories of the RumiWatchSystem were added up to a total eating time per hour. The location data from the Smartbow^®^ system of the cows within the defined area at the feed fence was already outputted as min/h per cow and could be analysed without further processing. The resulting data set contained 48 data points of all three systems for all 10 cows.

Two methods were then applied to examine the agreement of the different aspects of feeding behaviour. First, repeated measures correlations were calculated using the R package ‘rmcorr’ (rmcorr version 0.3.0, R version 3.6.2.; see also Bakdash and Marusich [22]). This was necessary, since the data contained repeated measurements of each cow. Second, two Bland–Altman plots were created to evaluate the absolute agreement between the UHF system and the other two sensor systems. In the Bland–Altman plots, the differences of two paired measurements of the two methods compared were plotted against the mean of these two measurements. In addition, the mean of all differences and limits of agreement calculated as the mean ± 1.96 × standard deviation of differences were added to the plots [23]. Normal distribution of the differences was checked using histograms. Assuming normal distribution, 95% of the differences lie within the upper and lower limits of agreement.

## 3. Results

### 3.1. Experiment 1: Setup and Test of the UHF RFID System

**Figure 6:**
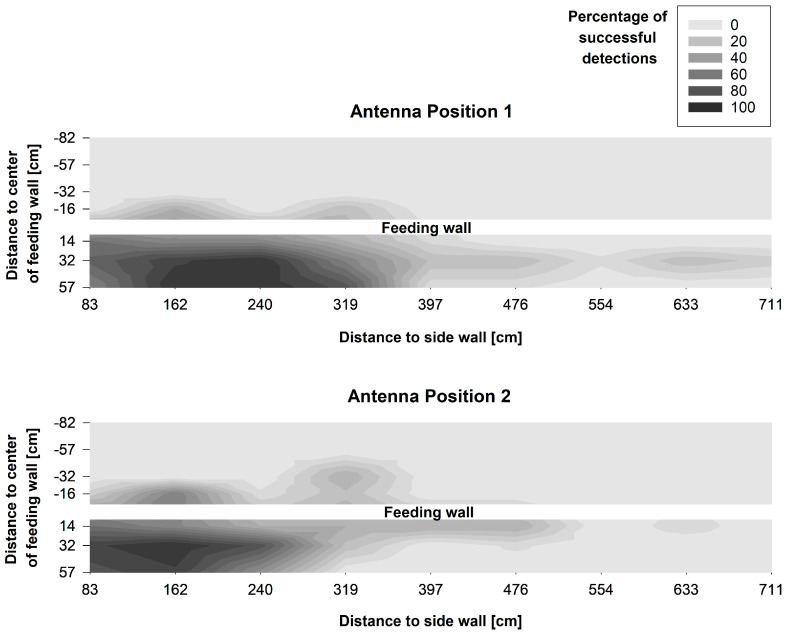
Interpolated contour plot showing the percentage of successful detections per coordinate of UHF ear tag Type A overall heights and for both antenna positions. Major ticks on the x- and y-axes indicate the measurement coordinates. Each x-coordinate represents the centre of a feeding place along the feed fence.

**Figure 7:**
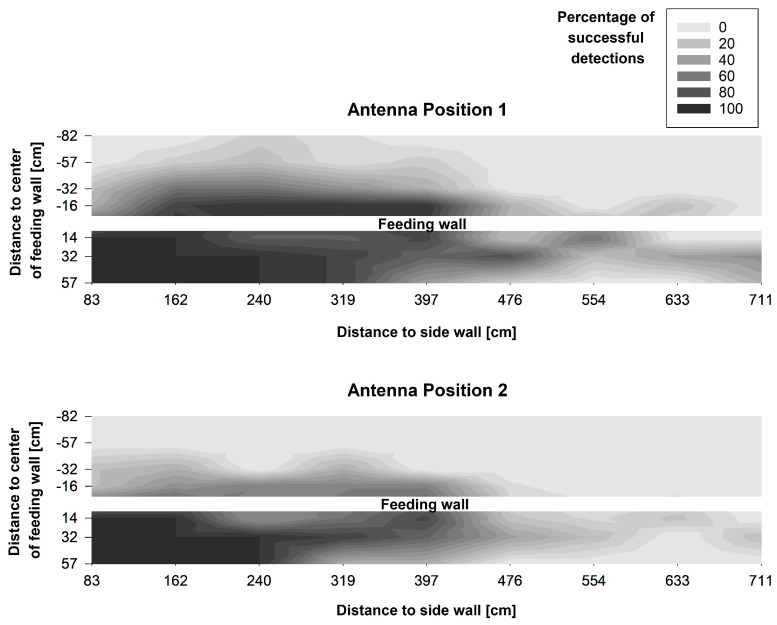
Interpolated contour plot showing the percentage of successful detections per coordinate of UHF ear tag Type B over all heights and for both antenna positions. Major ticks on the x- and y-axes indicate the measurement coordinates. Each x-coordinate represents the centre of a feeding place along the feed fence.

The visualisation of the measurements showed that the reading area differed between both antenna positions and also both types of UHF ear tag (Figure 6 and Figure 7). In all treatments, the reading area was not evenly shaped, but showed irregular gaps. In addition, sections with a higher reading range were also detected. Furthermore, a decrease in the reading range was visible over the length of the antenna, beginning with a higher detection rate and greater reading area at the tip of the antenna.

Tag Type B showed a higher detection rate than Type A, both in front and behind the feed fence. Furthermore, Tag B had a higher share of successful registrations than Tag A at Feeding Places 7, 8 and 9, which were positioned closer to the end of the antenna. With Antenna Position 1 more successful readings could be detected with both types of tag than with Antenna Position 2, resulting in a larger reading area with Antenna Positon 1. However, for ear tag Type A a slight increase in the detection rate behind the feed fence was registered with Antenna Position 2 in comparison to Antenna Position 1.

**Figure 8:**
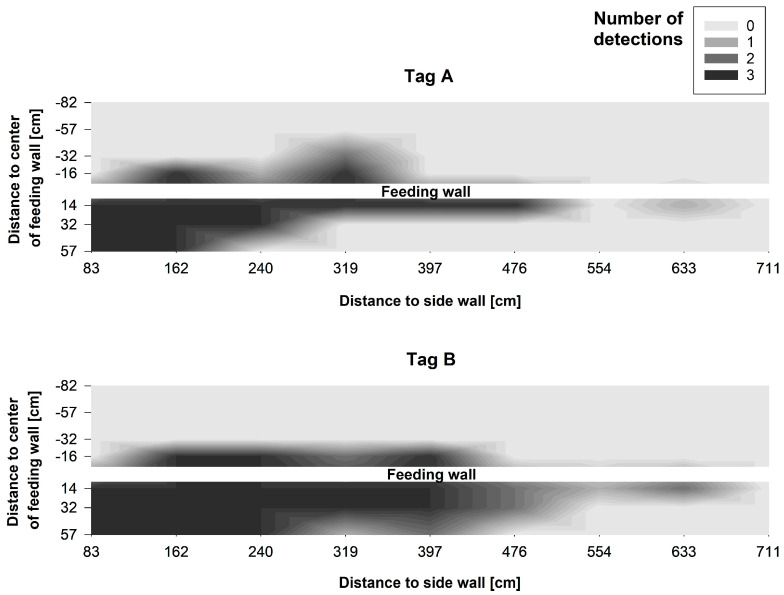
Interpolated contour plot showing the number of successful detections per coordinate for both types of UHF ear tag at a height of 55 cm and for Antenna Position 2. Major ticks on the x- and y-axes indicate the measurement coordinates. Each x-coordinate represents the centre of a feeding place along the feed fence.

Since most of the readings are expected to and should take place close to the floor when using the system to detect cows during feeding, a closer look was taken at the number of successful registrations at a height of 55 cm for Antenna Position 2 (Figure 8). It is visible that ear tag Type A was detectable without a gap from Feeding Place 1 to 6, and ear tag Type B from Feeding Place 1 to 8. At this height, both ear tags also showed readings behind the feed fence up to a distance of 57 cm (Type A) and 32 cm (Type B).

The differences between the antenna positions and the types of UHF ear tag were also evident in the number of coordinates, at which at least one successful detection of a tag was registered over all measurement heights. Focusing on unwanted registrations behind the feed fence, tag Type B was detected at 18 coordinates with Antenna Position 1 compared to only at 9 coordinates with Antenna Position 2. Interestingly, for tag Type A a slight increase from two coordinates in Antenna Position 1 to five coordinates in Antenna Position 2 behind the feed fence was registered. However, above 55 cm height, where cows position their head while standing in the walking alley, at only 6 coordinates 16 and 32 cm behind the feed fence readings were registered with Type B and only 2 with Type A.

In front of the feed fence, tag Type B was detected at 23 coordinates with Antenna Position 1 and at 21 coordinates with Antenna Position 2. Ear tag Type A was detected at 20 coordinates in front of the feed fence in Antenna Position 1 compared to 15 coordinates in Antenna Position 2.

### 3.2. Experiment 2: Validation of the UHF RFID System and Comparison to Two other Sensor Systems

The calculation of the overall accuracy for both types of tag for the combined video data set with the sub-events head up and head down resulted in an optimum bout criterion of 180 s. At this point the accuracy was highest and the numbers of false positives and false negatives were lowest. Increasing the bout criterion to 300 s did not improve the results further, but led to a lower accuracy due to a higher number of false positives. For the video visits only including the sub-events when the cows had their head down, the optimum bout criterion was 120 s.

The detailed results of the validation can be seen in Table 1. With both sub-events head up and head down analysed together the sums of false positives and false negatives were almost equal, while for the second dataset there were far more false positives, resulting in remarkably lower specificity and precision. The results for both types of tag were similar, with slightly higher sensitivity but lower precision for tag Type A. The total duration of the feeding visits of all 10 cows during the validation period of nearly 10 h was 25.02 h including both sub-events head up and head down and 18.95 h with only the sub-events head down, as extracted from the video data. The corresponding mean visiting time per cow was 2.50 ± 0.42 h (mean ± standard deviation; 1.89 ± 0.36 h with head down), ranging from 1.86 h to 3.09 h (1.51 to 2.52 h with head down). Consequently, the duration of non-events added up to a total of 74.08 h (80.16 h with head down). In total, 476 events were detected in the video data with the sub-events head up and head down together and 1810 with head down. The aggregated RFID data contained 75 events with a bout criterion of 180 s and 106 events with a bout criterion of 120 s.

During the 48 h of comparison between the UHF RFID system (both types of tag), the RumiWatch noseband sensor and the location data of the Smartbow^®^ system, the UHF system detected 94.13 h spent at the feed fence (9.41 ± 0.82 h per cow, mean ± standard deviation). The noseband sensor measured a total of 108.03 h eating time (10.8 ± 1.09 h per cow) and the location data resulted in a total of 69.75 h spent at the feed fence (6.98 ± 0.58 h per cow).

Despite these differences in absolute numbers, the repeated measures correlation coefficient for the 1-hour summaries of the UHF RFID data and the eating time detected by the noseband sensor was r = 0.95 (*p* < 0.001) and for the UHF RFID data and the time recorded by the real-time localisation r = 0.93 (*p* < 0.001).

**Figure 9:**
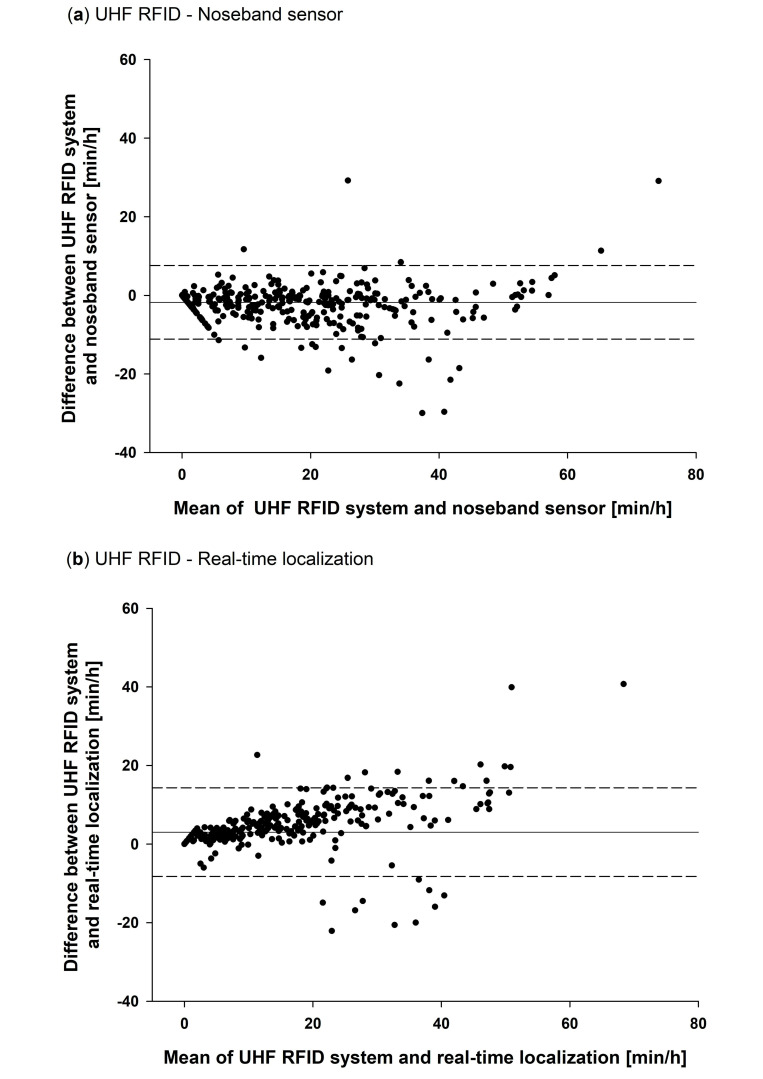
Agreement of the 1-hour summaries of time spent at the feed fence measured with the UHF RFID system versus (**a**) eating time of the RumiWatch noseband sensor and versus (**b**) the time spent at the feed fence extracted from the real-time localisation data of the Smartbow^®^ system shown in Bland–Altman plots. The solid line indicates the mean of the differences, the dashed lines indicate the upper and lower 95% limits of agreement.

The Blant–Altman analysis confirmed that the UHF RFID system on average measured a shorter time spent at the feed fence per hour than the eating time recorded with the noseband sensor (Figure 9a). The mean of the differences was −1.7 min/h with an upper limit of agreement of 7.6 min/hour and a lower limit of agreement of −11.1 min/h. In comparison with the location data, the UHF RFID system measured more time spent at the feed fence per hour (Figure 9b). The mean of the differences was 3.1 min/h with an upper limit of agreement of 14.3 min/hour and a lower limit of agreement of −8.2 min/h. A slight positive linearity visible in the Blant–Altman graph of the UHF RFID system and the Smartbow^®^ system suggests that the difference between these systems proportionally increased with increasing feeding duration, while the difference between the UHF RFID system and the noseband sensor was rather independent of the mean feeding duration.

## 4. Discussion

### 4.1. Experiment 1: Characterisation of the Reading Area and Suitability for the Targeted Application

The method applied for measuring the reading area in this experiment is rather simple compared to other methods of testing UHF RFID systems described by e.g., Derbek et al. [24] and Adrion et al. [25]. Thus, it is limited to the specific application environment of this experiment. However, the results were very consistent between all samples of each type of tag in both antenna positions. All things considered, this method seems to be a good compromise between the informative value of and effort for the measurements.

The results show the difficulty of generating an evenly shaped reading area with a UHF RFID system. Compared to low-frequency RFID systems, as presented for example by Brown-Brandl and Eigenberg [8], UHF RFID offers the possibility to use fewer antennas for the same number of feeding spaces, which could make such a system more cost-effective for commercial applications. Especially with the flex-form cable antennas used in this study, long reading areas can be covered. However, antenna fields always have a circular or elliptical shape, so that it is easier to adapt several small antenna fields to a rectangular reading area than few large fields, which was shown by Li et al. [11] and Brown-Brandl and Eigenberg [8]. In addition, the free-form cable antenna exhibits a decreasing extension of the reading field along the antenna. Hence, some overlap of the antennas was necessary to cover the whole length of the feed fence.

Antenna Position 2 was chosen for the setup of Experiment 2, because we put a stronger focus on avoiding false positive readings behind the feed fence. Antenna Position 1 showed more detections behind the feed fence, especially with tag Type B. Furthermore, above the 55 cm height, where cows typically position their head while standing in the walking alley, with Antenna Position 2 at only a few coordinates readings were registered behind the feed fence. In total, six to eight feeding spaces were reliably covered by both types of tag with Antenna Position 2, allowing the coverage of the whole feed fence with only three antennas in Experiment 2.

### 4.2. Experiment 2: Accuracy and Comparability of the UHF System

The validation results of the UHF RFID system with video data were comparable to those of other UHF RFID validation studies in recent publications. Accuracy was at an average of 96.9% for both head positions and 92.3% for head down. In comparison, Li et al. [11] found an overall accuracy of 92.1 ± 6.4% (mean ± SD) for a UHF RFID system measuring time laying hens spent with their head in the feeder. For pigs, Adrion et al. [10] achieved an accuracy between 96.5% and 98.7% for time spent at the feed trough with two different types of UHF antennas, including a cable antenna. However, sensitivity and precision (positive predictive value) should also always be taken into account, since a very high number of negatives in relation to a low number of positives increases specificity and, hence, total accuracy in most cases. This is due to animals being away from the target area most of the day, resulting in a high number of true negatives. Especially in pigs, feeding times can be as low as 40 to 60 min/d in certain feeding systems [10,26], which equals a relation of positives to negatives of 1:24 to 1:36. In contrast to this, this relation was only about 1:4 in for the cows in this study. While studies with pigs reported relatively low values of precision of only between 40% and 80% for RFID measurements of the time pigs spent at the feeder [10,26] still a high accuracy was achieved due to the high number of true negatives. In contrast to this, precision was 93.8% with visits including both head positions in this study. However, it was only 73.8% with visits including only head down. This was caused by, in this case, false positive registrations of the ear tags, while the cows held their heads up in front of the feed fence. This result shows that with the UHF RFID system the cows were detected in both head positions, so that both gathering of feed with the head down as well as mastication in an upward head position [27] could be detected.

The feed fence with its headlocks very likely contributed to the high precision due to less movement and displacements of cows compared to pigs at an open feeder [28]. In the feeding systems observed in Maselyne et al. [26] and Adrion et al. [10] pigs could easily displace each other causing a much higher number of individual visits and animals entering and leaving the reading field constantly. This possibly caused more false positives at the border of the target area. Furthermore, cows typically move slower than pigs, which makes it easier for an RFID system to detect their tags reliably inside the reading area.

The slightly higher sensitivity and lower specificity and precision of tag Type A indicate that in Experiment 2 the readability of tag Type B was inferior. These results are in contrast to the measurements of reading range in Experiment 1, where Type B showed a higher reading performance. A very likely explanation of this effect is a better adaption of Type A to the vicinity of ear tissue. Since body tissue has a high loss tangent and a high relative permittivity, it has a negative effect on the reading range of UHF RFID transponders, mostly due to energy losses and a change of the transponder antenna impedance [29]. Targeted design and detuning of the transponder antenna can decrease the effect of the impedance change and thus increase the performance of a transponder when being used on animals [14]. In this case, tag Type A was probably adapted better to the vicinity of ear tissue than Type B.

The bout criteria of 180 s and 120 s chosen in this study were much higher than in other studies detecting time spent at the feeder with RFID systems, where bout criteria were between 10 s and 70 s [10,11,26,30]. Consequently, the number of visiting events was much lower in the RFID data than in the (not aggregated) video data, so that e.g., fast changes of cows between feeding places were not represented as single events in the aggregated RFID data. The high bout criteria also shows that the gaps between single registrations of the tags must have been higher in this study compared to the other studies. A probable explanation for this is the ear tag position within the cows’ ears. In this study, the part of the ear tag containing the transponder was in front of the ear while the reader antenna was behind the head and ear of the cows. Thus, the ear tissue probably absorbed part of the radiation from the reader antenna in many positions of the cows’ heads, leading to reading gaps until the tag was readable again after a movement of the cow [14].

The highly significant correlation of the hourly time spent at the feed fence measured with the UHF RFID system and the eating time measured with the RumiWatch noseband pressure sensor as well as the time spent at the feed fence extracted from the location data of the Smartbow^®^ system indicate that the UHF data is generally suitable for measuring feeding time of cows. However, in absolute numbers, considerable average differences between the measurements of the three systems are obvious and also some strong outliers were detected (Figure 9). This is most likely due to the different aspects of feeding behaviour represented by the data of the three systems.

It can be assumed that measuring the jaw movements of the animals with the noseband pressure sensor is the most direct and, hence, most accurate measure of eating time of the three systems compared. Since the pressure sensor is mounted on the animal, it can measure eating at all times and any place in the barn, while both the UHF RFID system and the real-time localisation only record the time spent at the feed fence. Especially the eating time in a concentrate feeding station was not measured by the two other systems, probably explaining some of the strong negative outliers in Figure 9a and the average longer feeding durations measured with the noseband pressure sensor. Positive differences between the UHF RFID system and the noseband sensor, which also occurred, were most likely caused by cows which were registered at the feed fence, but did not actually eat.

The average shorter time spent at the feed fence recorded with the real-time localisation of the Smartbow^®^ system can possibly be explained with the position error of this system of a minimum of 1.2 m, measured by Wolfger et al. [21]. Due to disturbance of the signals sent from the tags to the receivers in the barn by reflections or blockage, localisation errors can occur regularly in such a system. Especially at the feed fence made of metal, this could have caused position estimates out of the target area, while the cows were standing at the feed fence. Of course, also cows standing close to the feed fence could have been registered within the target area by the real-time localisation causing longer measurements than detected with the UHF RFID system, which are also visible in Figure 9b.

The aggregation of the UHF RFID data with a bout criterion could also have caused part of the difference between the systems. If cows changed their feeding place within a time below the bout criterion, these single visits were combined in the UHF RFID data, resulting in longer time at the feed fence measured with the UHF RFID system than with the real-time localisation and maybe also with the noseband sensor. Furthermore, false positive measurements of the UHF RFID system could occur when a cow was registered behind the feed fence. On the other hand, visits might have been missed by the system at zones in the reading field, where the field was weaker due to destructive interference of reflected waves of the reader antenna [16].

In summary, the good correlation but only partially good agreement of the different measures of feeding behaviour from the three technologies point out differences between direct and indirect measures of feeding behaviour in general and between different sensors in particular. Thus, detailed considerations are necessary before interpreting automatically measured feeding data generally. It is important to determine the purpose of the expected data before choosing and implementing a sensor system. Furthermore, this study shows that the performance of UHF RFID systems in animal monitoring is partially limited due to the physical properties of the radiation in the UHF frequency band. Secondly, all passive RFID systems are only designed to detect the presence of tags, but not their exact position within the reading area. This could be improved by further developments such as combined passive ultra-wideband (UWB) UHF RFID tags. By using UWB signals next to the UHF band in one tag, time-based localisation can be implemented [31]. In this way, unwanted registrations in certain areas of the reading field could be filtered and additional information could be extracted from the location measurements.

## 5. Conclusions

The results of this study point out the challenges of setting up UHF RFID antennas in a barn environment. Especially with the free-form cable antenna used, the influence of metal in the surrounding area was evident. Thus, the results confirm that thorough testing is necessary for new application environments of UHF RFID systems. However, UHF RFID still offers greater flexibility for monitoring animal behaviour in various applications compared to other RFID frequencies. The validation of the tested UHF RFID system with video data showed a high accuracy and precision of the detection of cows’ feeding visits. The measurements were also significantly correlated with the eating time data of a noseband pressure sensor and the position data of a sensor system with real-time localisation, but with differences in absolute duration. In conclusion, the use of this UHF RFID system in a commercial setting is promising. However, such a system records the time spent at the feed fence; it registers neither the actual eating time nor the feed intake. This must be taken into account when interpreting data on farms.

## Figures and Tables

**Table 1:** Mean ± standard deviation of sensitivity, specificity, precision and accuracy as well as number of false positives and false negatives of the UHF radio-frequency identification (RFID) data validated with two reference data sets of video events with cows (**a**) having their head up and down in front of the feed fence or (**b**) only head down.

Video Data	Bout Criterion (s)	Tag Type	Sensitivity (%)	Specificity (%)	Precision (%)	Accuracy (%)	False Positives	False Negatives
Head up + down	180	A + B	93.7 ± 5.6	97.8 ± 1.1	93.8 ± 2.3	96.9 ± 0.9	5778	5110
		A	95.7 ± 3.7	97.5 ± 1.0	93.3 ± 1.5	97.2 ± 0.8	3247	1765
		B	91.7 ± 6.8	98.1 ± 1.2	94.3 ± 2.8	96.7 ± 0.8	2531	3345
Head down	120	A + B	92.5 ± 5.2	92.1 ± 2.8	73.8 ± 5.7	92.3 ± 2.1	22,751	4690
		A	94.3 ± 3.5	91.1 ± 3.2	72.7 ± 5.9	91.8 ± 2.5	12,713	1853
		B	90.6 ± 6.3	93.0 ± 2.1	74.9 ± 5.9	92.8 ± 1.9	10,038	2837
